# Supplementary figures and images for: Survival of HT29 cancer cells is influenced by hepatocyte growth factor receptor inhibition through modulation of self-DNA-triggered TLR9-dependent autophagy response
Source: PLoS One. 2022 May 12;17(5):e0268217. doi: 10.1371/journal.pone.0268217 (PMC9098092; doi:10.1371/journal.pone.0268217)

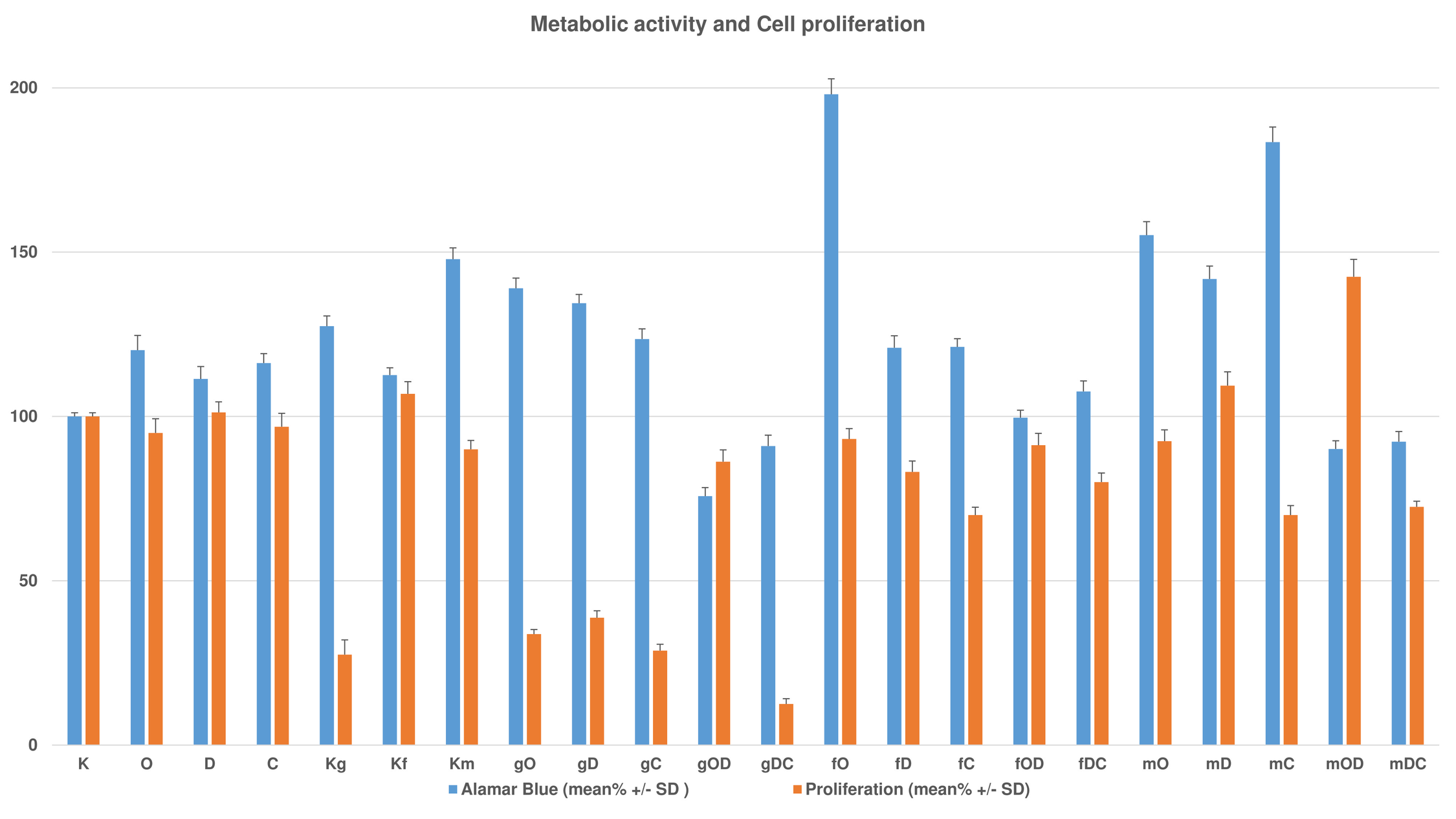

Supplement: S1 Fig — Changes in the metabolic activity (blue) and proliferation (orange) of the studied cell groups under the influence of each treatment combination. The red star indicates the lowest (group gDC), while the red triangle indicates the highest proliferative activity (mOD group). g/f/mDNA: genomic/fragmented/hypermethylated deoxyribonucleic acid; ODN: CpG oligonucleotide; DISU: 4,4’Diisothiocyanatostilbene-2,2’-disulfonic acid; C: chloroquine; SD: standard deviation. (TIF) [file pone.0268217.s001.tif]

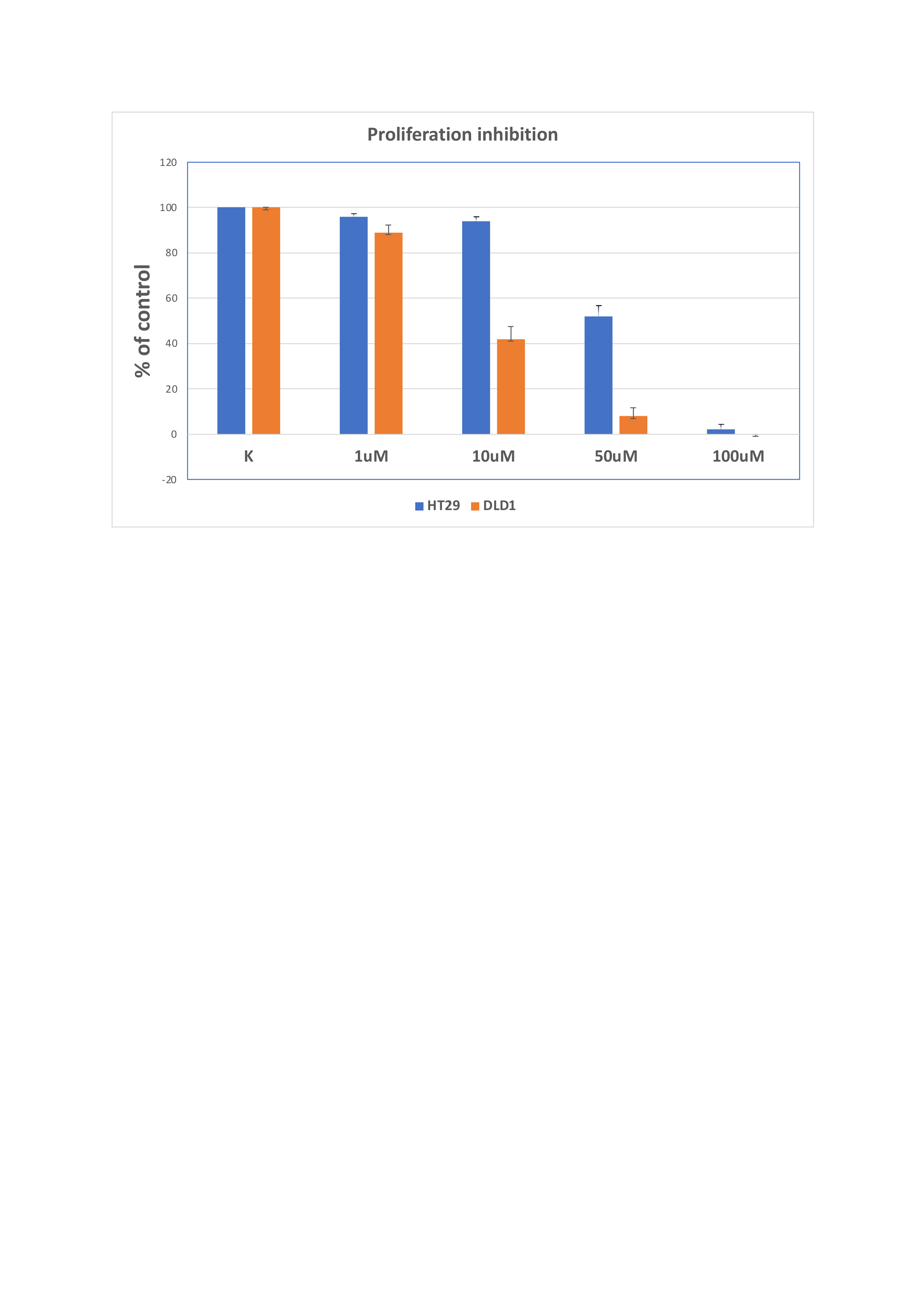

Supplement: S2 Fig — Based on our preliminary experiments, chloroquine treatment at a concentration of 10 μM effectively inhibited autophagy without significantly affecting the proliferation of HT29 cells after 72h of incubation. (TIF) [file pone.0268217.s002.tif]
