# Supplementary material for: Survival of HT29 cancer cells is influenced by hepatocyte growth factor receptor inhibition through modulation of self-DNA-triggered TLR9-dependent autophagy response
Source: PLoS One. 2022 May 12;17(5):e0268217. doi: 10.1371/journal.pone.0268217 (PMC9098092; doi:10.1371/journal.pone.0268217)
Supplement: S1 Table — Regarding HT29 cell viability and cell number data, the results of the statistical analyses with one-way ANOVA and Tukey HSD test are displayed. (PDF) [file pone.0268217.s003.pdf]

Supplementary Table 1.

	AlamarBlue	SD
K	100	1,1
O	120,17	4,5
D	111,41	3,8
C	116,23	2,9
Kg	127,51	3,1
Kf	112,61	2,2
Km	147,87	3,4
gO	139	3,1
gD	134,44	2,7
gC	123,55	3,1
gOD	75,75	2,6
gDC	90,99	3,3
fO	198,02	4,7
fD	120,87	3,7
fC	121,18	2,5
fOD	99,61	2,3
fDC	107,62	3,2
mO	155,15	4,1
mD	141,85	3,9
mC	183,48	4,6
mOD	90,12	2,5
mDC	92,34	3,1

Descriptive statistics of the treatments:								
**Treatment →**	**K**	**O**	**D**	**C**	**Kg**	**Kf**	**Km**	Pooled Total
observations N	3	3	3	3	3	3	3	21
sum ∑xiΣxi	300.0000	360.5100	334.2300	348.6900	382.5300	337.8300	443.6100	2,507.4000
mean ¯xx¯	100.0000	120.1700	111.4100	116.2300	127.5100	112.6100	147.8700	119.4000
sum of squares ∑x2iΣxi2	30,002.4200	43,362.9867	37,265.4443	40,545.0587	48,795.6203	38,052.7163	65,619.7307	303,643.9770
sample variance s2s2	1.2100	20.2500	14.4400	8.4100	9.6100	4.8400	11.5600	213.0209
sample std. dev. ss	1.1000	4.5000	3.8000	2.9000	3.1000	2.2000	3.4000	14.5952
std. dev. of mean SE¯xSEx¯	0.6351	2.5981	2.1939	1.6743	1.7898	1.2702	1.9630	3.1849
One-way ANOVA of the treatments:								
source	sum of	degrees of	mean square	F statistic	**p-value**			
	squares SS	freedom νv	MS					
treatment	4,119.7770	6	686.6295	68.3505	**1.4503e-09**			
error	140.6400	14	10.0457					
total	4,260.4170	20						
**Tukey HSD results**								
treatments	Tukey HSD	**Tukey HSD**	**Tukey HSD**					
pair	Q statistic	**p-value**	**inferfence**					
**K vs O**	11.0224	**0.0010053**	**** p<0.01**					
**K vs D**	6.2353	**0.0082450**	**** p<0.01**					
**K vs C**	8.8693	**0.0010053**	**** p<0.01**					
**K vs Kg**	15.0335	**0.0010053**	**** p<0.01**					
**K vs Kf**	6.8910	**0.0035694**	**** p<0.01**					
**K vs Km**	26.1598	**0.0010053**	**** p<0.01**					
**O vs D**	4.7871	**0.0527418**	**insignificant**					
**O vs C**	2.1531	**0.7068347**	**insignificant**					
**O vs Kg**	4.0111	**0.1353000**	**insignificant**					
**O vs Kf**	4.1314	**0.1177960**	**insignificant**					
**O vs Km**	15.1374	**0.0010053**	**** p<0.01**					
**D vs C**	2.6340	**0.5291763**	**insignificant**					
**D vs Kg**	8.7982	**0.0010053**	**** p<0.01**					
**D vs Kf**	0.6558	**0.8999947**	**insignificant**					
**D vs Km**	19.9245	**0.0010053**	**** p<0.01**					
**C vs Kg**	6.1642	**0.0090323**	**** p<0.01**					
**C vs Kf**	1.9782	**0.7714362**	**insignificant**					
**C vs Km**	17.2905	**0.0010053**	**** p<0.01**					
**Kg vs Kf**	8.1425	**0.0010053**	**** p<0.01**					
**Kg vs Km**	11.1262	**0.0010053**	**** p<0.01**					
**Kf vs Km**	19.2687	**0.0010053**	**** p<0.01**					

Descriptive statistics of the treatments:							
**Treatment →**	**K**	**gO**	**gD**	**gC**	**gOD**	**gDC**	Pooled Total
observations N	3	3	3	3	3	3	18
sum ∑xiΣxi	300.0000	417.0000	403.3200	370.6500	227.2800	272.9700	1,991.2200
mean ¯xx¯	100.0000	139.0000	134.4400	123.5500	75.7600	90.9900	110.6233
sum of squares ∑x2iΣxi2	30,002.4200	57,982.2200	54,236.9208	45,813.0275	17,232.4094	24,859.3203	230,126.3180
sample variance s2s2	1.2100	9.6100	7.2900	9.6100	6.8383	10.8900	579.4661
sample std. dev. ss	1.1000	3.1000	2.7000	3.1000	2.6150	3.3000	24.0721
std. dev. of mean SE¯xSEx¯	0.6351	1.7898	1.5588	1.7898	1.5098	1.9053	5.6738
One-way ANOVA of the treatments:							
source	sum of squares SS	degrees of freedom νν	mean square MS	F statistic	**p-value**		
treatment	9,760.0276	5	1,952.0055	257.7001	**8.9428e-12**		
error	90.8966	12	7.5747				
total	9,850.9242	17					
**Tukey HSD results**							
treatments pair	Tukey HSD Q statistic	**Tukey HSD p-value**	**Tukey HSD inferfence**				
**K vs gO**	24.5438	**0.0010053**	**** p<0.01**				
**K vs gD**	21.6741	**0.0010053**	**** p<0.01**				
**K vs gC**	14.8207	**0.0010053**	**** p<0.01**				
**K vs gOD**	15.2549	**0.0010053**	**** p<0.01**				
**K vs gDC**	5.6703	**0.0167034**	*** p<0.05**				
**gO vs gD**	2.8697	**0.3825024**	**insignificant**				
**gO vs gC**	9.7231	**0.0010053**	**** p<0.01**				
**gO vs gOD**	39.7987	**0.0010053**	**** p<0.01**				
**gO vs gDC**	30.2141	**0.0010053**	**** p<0.01**				
**gD vs gC**	6.8534	**0.0041561**	**** p<0.01**				
**gD vs gOD**	36.9290	**0.0010053**	**** p<0.01**				
**gD vs gDC**	27.3443	**0.0010053**	**** p<0.01**				
**gC vs gOD**	30.0756	**0.0010053**	**** p<0.01**				
**gC vs gDC**	20.4909	**0.0010053**	**** p<0.01**				
**gOD vs gDC**	9.5847	**0.0010053**	**** p<0.01**				

Descriptive statistics of the treatments:							
**Treatment →**	**K**	**fO**	**fD**	**fC**	**fOD**	**fDC**	Pooled Total
observations N	3	3	3	3	3	3	18
sum ∑xiΣxi	300.0000	594.0600	362.6100	363.5400	298.8300	322.8600	2,241.9000
mean ¯xx¯	100.0000	198.0200	120.8700	121.1800	99.6100	107.6200	124.5500
sum of squares ∑x2iΣxi2	30,002.4200	117,679.9412	43,856.0507	44,066.2772	29,777.0363	34,766.6732	300,148.3986
sample variance s2s2	1.2100	22.0900	13.6900	6.2500	5.2900	10.2400	1,230.5737
sample std. dev. ss	1.1000	4.7000	3.7000	2.5000	2.3000	3.2000	35.0795
std. dev. of mean SE¯xSEx¯	0.6351	2.7135	2.1362	1.4434	1.3279	1.8475	8.2683
One-way ANOVA of the treatments:							
source	sum of squares SS	degrees of freedom νν	mean square MS	F statistic	**p-value**		
treatment	20,802.2136	5	4,160.4427	424.7517	**4.5797e-13**		
error	117.5400	12	9.7950				
total	20,919.7536	17					
**Tukey HSD results**							
treatments pair	Tukey HSD Q statistic	**Tukey HSD p-value**	**Tukey HSD inferfence**				
**K vs fO**	54.2467	**0.0010053**	**** p<0.01**				
**K vs fD**	11.5500	**0.0010053**	**** p<0.01**				
**K vs fC**	11.7215	**0.0010053**	**** p<0.01**				
**K vs fOD**	0.2158	**0.8999947**	**insignificant**				
**K vs fDC**	4.2171	**0.0932784**	**insignificant**				
**fO vs fD**	42.6967	**0.0010053**	**** p<0.01**				
**fO vs fC**	42.5251	**0.0010053**	**** p<0.01**				
**fO vs fOD**	54.4625	**0.0010053**	**** p<0.01**				
**fO vs fDC**	50.0296	**0.0010053**	**** p<0.01**				
**fD vs fC**	0.1716	**0.8999947**	**insignificant**				
**fD vs fOD**	11.7658	**0.0010053**	**** p<0.01**				
**fD vs fDC**	7.3329	**0.0024090**	**** p<0.01**				
**fC vs fOD**	11.9374	**0.0010053**	**** p<0.01**				
**fC vs fDC**	7.5044	**0.0019876**	**** p<0.01**				
**fOD vs fDC**	4.4329	**0.0727690**	**insignificant**				

Descriptive statistics of the treatments:							
**Treatment →**	**K**	**mO**	**mD**	**mC**	**mOD**	**mDC**	Pooled Total
observations N	3	3	3	3	3	3	18
sum ∑xiΣxi	300.0000	465.4500	425.5500	550.4400	270.3600	277.0200	2,288.8200
mean ¯xx¯	100.0000	155.1500	141.8500	183.4800	90.1200	92.3400	127.1567
sum of squares ∑x2iΣxi2	30,002.4200	72,248.1875	60,394.6875	101,037.0512	24,377.3432	25,599.2468	313,658.9362
sample variance s2s2	1.2100	16.8100	15.2100	21.1600	6.2500	9.6100	1,330.6008
sample std. dev. ss	1.1000	4.1000	3.9000	4.6000	2.5000	3.1000	36.4774
std. dev. of mean SE¯xSEx¯	0.6351	2.3671	2.2517	2.6558	1.4434	1.7898	8.5978
One-way ANOVA of the treatments:							
source	sum of	degrees of	mean square	F statistic	**p-value**		
	squares SS	freedom νv	MS				
treatment	22,479.7144	5	4,495.9429	383.9951	**8.3522e-13**		
error	140.5000	12	11.7083				
total	22,620.2144	17					
**Tukey HSD results**							
treatments	Tukey HSD	**Tukey HSD**	**Tukey HSD**				
pair	Q statistic	**p-value**	**inferfence**				
**K vs mO**	27.9163	**0.0010053**	**** p<0.01**				
**K vs mD**	21.1840	**0.0010053**	**** p<0.01**				
**K vs mC**	42.2567	**0.0010053**	**** p<0.01**				
**K vs mOD**	5.0012	**0.0371402**	*** p<0.05**				
**K vs mDC**	3.8774	**0.1369165**	**insignificant**				
**mO vs mD**	6.7323	**0.0047827**	**** p<0.01**				
**mO vs mC**	14.3403	**0.0010053**	**** p<0.01**				
**mO vs mOD**	32.9175	**0.0010053**	**** p<0.01**				
**mO vs mDC**	31.7938	**0.0010053**	**** p<0.01**				
**mD vs mC**	21.0727	**0.0010053**	**** p<0.01**				
**mD vs mOD**	26.1852	**0.0010053**	**** p<0.01**				
**mD vs mCD**	25.0614	**0.0010053**	**** p<0.01**				
**mC vs mOD**	47.2578	**0.0010053**	**** p<0.01**				
**mC vs mDC**	46.1341	**0.0010053**	**** p<0.01**				
**mOD vs mDC**	1.1237	**0.8999947**	**insignificant**				

	**Average cell number**	**SD**
**K**	800000	8801
**O**	760000	32681
**D**	810000	25921
**C**	775000	31776
**Kg**	220000	9901
**Kf**	855000	31636
**Km**	720000	19441
**gO**	270000	3781
**gD**	310000	6511
**gC**	230000	4371
**gOD**	690000	24841
**gDC**	100000	1601
**fO**	745000	23841
**fD**	665000	21946
**fC**	560000	13441
**fOD**	730000	26281
**fDC**	640000	17921
**mO**	740000	25161
**mD**	875000	36751
**mC**	560000	16241
**mOD**	1140000	60421
**mDC**	580000	9861

Descriptive statistics of the treatments:							
**Treatment →**	**K**	**O**	**D**	**C**	**Kg**	**Kf**	**Km**	Pooled Total
observations N	3	3	3	3	3	3	3	21
sum ∑xi∑xi	2,400,000.0000	2,280,000.0000	2,430,000.0000	2,325,000.0000	660,000.0000	2,565,000.0000	2,160,000.0000	14,820,000.0000
mean ¯xx¯	800,000.0000	760,000.0000	810,000.0000	775,000.0000	220,000.0000	855,000.0000	720,000.0000	705,714.2857
sum of squares ∑x2i∑xi2	1,920,154,915,	1,734,936,095,	1,969,643,796,	1,803,894,428,	145,396,059,60	2,195,076,672,	1,555,955,904,962.00	11,325,057,873,114.0000
sample variance s2s2	77,457,601.000	1,068,047,761.	671,898,241.00	1,009,714,176.	98,029,801.000	1,000,836,496.	377,952,481.0000	43,318,607,941.4143
sample std. dev. ss	8,801.0000	32,681.0000	25,921.0000	31,776.0000	9,901.0000	31,636.0000	19,441.0000	208,131.2277
std. dev. of mean SE¯xSEx¯	5,081.2597	18,868.3841	14,965.4963	18,345.8822	5,716.3450	18,265.0531	11,224.2666	45,417.9574
One-way ANOVA of the treatments:								
source	sum of	degrees of	mean square	F statistic	**p-value**			
	squares SS	freedom νv	MS					
treatment	857,764,285,71	6	142,960,714,28	232.5139	**3.3806e-13**			
error	8,607,873,114.	14	614,848,079.5714					
total	866,372,158,82	20						
**Tukey HSD results**								
treatments	Tukey HSD	**Tukey HSD**	**Tukey HSD**					
pair	Q statistic	**p-value**	**inferfence**					
**K vs O**	2.7941	**0.4698752**	**insignificant**					
**K vs D**	0.6985	**0.8999947**	**insignificant**					
**K vs C**	1.7463	**0.8571221**	**insignificant**					
**K vs Kg**	40.5140	**0.0010053**	**** p<0.01**					
**K vs Kf**	3.8418	**0.1646564**	**insignificant**					
**K vs Km**	5.5881	**0.0189808**	*** p<0.05**					
**O vs D**	3.4926	**0.2412018**	**insignificant**					
**O vs C**	1.0478	**0.8999947**	**insignificant**					
**O vs Kg**	37.7199	**0.0010053**	**** p<0.01**					
**O vs Kf**	6.6359	**0.0049383**	**** p<0.01**					
**O vs Km**	2.7941	**0.4698752**	**insignificant**					
**D vs C**	2.4448	**0.5990719**	**insignificant**					
**D vs Kg**	41.2125	**0.0010053**	**** p<0.01**					
**D vs Kf**	3.1433	**0.3432522**	**insignificant**					
**D vs Km**	6.2866	**0.0077161**	**** p<0.01**					
**C vs Kg**	38.7677	**0.0010053**	**** p<0.01**					
**C vs Kf**	5.5881	**0.0189808**	*** p<0.05**					
**C vs Km**	3.8418	**0.1646564**	**insignificant**					
**Kg vs Kf**	44.3558	**0.0010053**	**** p<0.01**					
**Kg vs Km**	34.9258	**0.0010053**	**** p<0.01**					
**Kf vs Km**	9.4300	**0.0010053**	**** p<0.01**					

Descriptive statistics of the treatments:							
**Treatment →**	**K**	**gO**	**gD**	**gC**	**gOD**	**gDC**	Pooled Total
observations N	3	3	3	3	3	3	18
sum ∑xi∑xi	2,400,000.0000	810,000.0000	930,000.0000	690,000.0000	2,070,000.0000	300,000.0000	7,200,000.0000
mean ¯xx¯	800,000.0000	270,000.0000	310,000.0000	230,000.0000	690,000.0000	100,000.0000	400,000.0000
sum of squares ∑x2i∑xi2	1,920,154,915,	218,728,591,92	288,384,786,24	158,738,211,28	1,429,534,150,	30,005,126,402	4,045,545,781,612.00
sample variance s2s2	77,457,601.000	14,295,961.000	42,393,121.000	19,105,641.000	617,075,281.00	2,563,201.0000	68,561,516,565.4118
sample std. dev. ss	8,801.0000	3,781.0000	6,511.0000	4,371.0000	24,841.0000	1,601.0000	261,842.5416
std. dev. of mean SE¯xSEx¯	5,081.2597	2,182.9614	3,759.1276	2,523.5980	14,341.9580	924.3378	61,716.8789
One-way ANOVA of the treatments:							
source+H56:H80	sum of	degrees of	mean square	F statistic	**p-value**		
	squares SS	freedom νv	MS				
treatment	1,164,000,000,	5	232,800,000,00	1,807.2411	**1.1102e-16**		
error	1,545,781,612.	12	128,815,134.3333				
total	1,165,545,781,	17					
**Tukey HSD results**							
treatments	Tukey HSD	**Tukey HSD**	**Tukey HSD**				
pair	Q statistic	**p-value**	**inferfence**				
**K vs gO**	80.8822	**0.0010053**	**** p<0.01**				
**K vs gD**	74.7779	**0.0010053**	**** p<0.01**				
**K vs gC**	86.9865	**0.0010053**	**** p<0.01**				
**K vs gOD**	16.7869	**0.0010053**	**** p<0.01**				
**K vs gDC**	106.8256	**0.0010053**	**** p<0.01**				
**gO vs gD**	6.1043	**0.0099746**	**** p<0.01**				
**gO vs gC**	6.1043	**0.0099746**	**** p<0.01**				
**gO vs gOD**	64.0953	**0.0010053**	**** p<0.01**				
**gO vs gDC**	25.9434	**0.0010053**	**** p<0.01**				
**gD vs gC**	12.2086	**0.0010053**	**** p<0.01**				
**gD vs gOD**	57.9910	**0.0010053**	**** p<0.01**				
**gD vs gDC**	32.0477	**0.0010053**	**** p<0.01**				
**gC vs gOD**	70.1997	**0.0010053**	**** p<0.01**				
**gC vs gDC**	19.8390	**0.0010053**	**** p<0.01**				
**gOD vs gDC**	90.0387	**0.0010053**	**** p<0.01**				

Descriptive statistics of the treatments:							
**Treatment →**	**K**	**fO**	**fD**	**fC**	**fOD**	**fDC**	Pooled Total
observations N	3	3	3	3	3	3	18
sum ∑xiΣxi	2,400,000.0000	2,235,000.0000	1,995,000.0000	1,680,000.0000	2,190,000.0000	1,920,000.0000	12,420,000.0000
mean ¯xx¯	800,000.0000	745,000.0000	665,000.0000	560,000.0000	730,000.0000	640,000.0000	690,000.0000
sum of squares ∑x2iΣxi2	1,920,154,915,	1,666,211,786,	1,327,638,253,	941,161,320,96	1,600,081,381,	1,229,442,324,	8,684,689,982,962.00
sample variance s2s2	77,457,601.000	568,393,281.00	481,626,916.00	180,660,481.00	690,690,961.00	321,162,241.00	6,758,234,291.8824
sample std. dev. ss	8,801.0000	23,841.0000	21,946.0000	13,441.0000	26,281.0000	17,921.0000	82,208.4807
std. dev. of mean SE¯xSEx¯	5,081.2597	13,764.6078	12,670.5290	7,760.1650	15,173.3424	10,346.6942	19,376.7247
One-way ANOVA of the treatments:							
source	sum of	degrees of	mean square	F statistic	**p-value**		
	squares SS	freedom νv	MS				
treatment	110,250,000,00	5	22,050,000,000	57.0261	**6.0351e-08**		
error	4,639,982,962.	12	386,665,246.8333				
total	114,889,982,96	17					
**Tukey HSD results**							
treatments	Tukey HSD	**Tukey HSD**	**Tukey HSD**				
pair	Q statistic	**p-value**	**inferfence**				
**K vs fO**	4.8446	**0.0447549**	*** p<0.05**				
**K vs fD**	11.8912	**0.0010053**	**** p<0.01**				
**K vs fC**	21.1400	**0.0010053**	**** p<0.01**				
**K vs fOD**	6.1658	**0.0092762**	**** p<0.01**				
**K vs fDC**	14.0933	**0.0010053**	**** p<0.01**				
**fO vs fD**	7.0467	**0.0033320**	**** p<0.01**				
**fO vs fC**	16.2954	**0.0010053**	**** p<0.01**				
**fO vs fOD**	1.3212	**0.8999947**	**insignificant**				
**fO vs fDC**	9.2487	**0.0010053**	**** p<0.01**				
**fD vs fC**	9.2487	**0.0010053**	**** p<0.01**				
**fD vs fOD**	5.7254	**0.0156417**	*** p<0.05**				
**fD vs fDC**	2.2021	**0.6252415**	**insignificant**				
**fC vs fOD**	14.9741	**0.0010053**	**** p<0.01**				
**fC vs fDC**	7.0467	**0.0033320**	**** p<0.01**				
**fOD vs fDC**	7.9275	**0.0012432**	**** p<0.01**				

Descriptive statistics of the treatments:							
**Treatment →**	**K**	**mO**	**mD**	**mC**	**mOD**	**mDC**	Pooled Total
observations N	3	3	3	3	3	3	18
sum ∑xiΣxi	2,400,000.0000	2,220,000.0000	2,625,000.0000	1,680,000.0000	3,420,000.0000	1,740,000.0000	14,085,000.0000
mean ¯xx¯	800,000.0000	740,000.0000	875,000.0000	560,000.0000	1,140,000.0000	580,000.0000	782,500.0000
sum of squares ∑x2iΣxi2	1,920,154,915,	1,644,066,151,	2,299,576,272,	941,327,540,16	3,906,101,394,	1,009,394,478,	11,720,620,752,332.0
sample variance s2s2	77,457,601.000	633,075,921.00	1,350,636,001.	263,770,081.00	3,650,697,241.	97,239,321.000	41,124,014,843.0588
sample std. dev. ss	8,801.0000	25,161.0000	36,751.0000	16,241.0000	60,421.0000	9,861.0000	202,790.5689
std. dev. of mean SE¯xSEx¯	5,081.2597	14,526.7101	21,218.1997	9,376.7457	34,884.0806	5,693.2510	47,798.1955
One-way ANOVA of the treatments:							
source	sum of	degrees of	mean square	F statistic	**p-value**		
	squares SS	freedom νv	MS				
treatment	686,962,500,00	5	137,392,500,00	135.7438	**3.9421e-10**		
error	12,145,752,332	12	1,012,146,027.6667				
total	699,108,252,33	17					
**Tukey HSD results**							
treatments	Tukey HSD	**Tukey HSD**	**Tukey HSD**				
pair	Q statistic	**p-value**	**inferfence**				
**K vs mO**	3.2666	**0.2618588**	**insignificant**				
**K vs mD**	4.0832	**0.1088206**	**insignificant**				
**K vs mC**	13.0662	**0.0010053**	**** p<0.01**				
**K vs mOD**	18.5105	**0.0010053**	**** p<0.01**				
**K vs mDC**	11.9774	**0.0010053**	**** p<0.01**				
**mO vs mD**	7.3498	**0.0023643**	**** p<0.01**				
**mO vs mC**	9.7997	**0.0010053**	**** p<0.01**				
**mO vs mOD**	21.7770	**0.0010053**	**** p<0.01**				
**mO vs mDC**	8.7108	**0.0010053**	**** p<0.01**				
**mD vs mC**	17.1494	**0.0010053**	**** p<0.01**				
**mD vs mOD**	14.4273	**0.0010053**	**** p<0.01**				
**mD vs mDC**	16.0606	**0.0010053**	**** p<0.01**				
**mC vs mOD**	31.5767	**0.0010053**	**** p<0.01**				
**mC vs mDC**	1.0889	**0.8999947**	**insignificant**				
**mOD vs mDC**	30.4879	**0.0010053**	**** p<0.01**				
