# Supplementary material for: Survival of HT29 cancer cells is influenced by hepatocyte growth factor receptor inhibition through modulation of self-DNA-triggered TLR9-dependent autophagy response
Source: PLoS One. 2022 May 12;17(5):e0268217. doi: 10.1371/journal.pone.0268217 (PMC9098092; doi:10.1371/journal.pone.0268217)
Supplement: Attachment — Submitted filename: Response to Reviewers.docx [file pone.0268217.s004.docx]

**Dear Editor-in-Chief,**

**Dear Editorial Office,**

We respectfully submit the improved version of our manuscript, entitled „Survival of HT29 cancer cells is influenced by hepatocyte growth factor receptor inhibition through modulation of self-DNA-triggered TLR9-dependent autophagy response”.

Thank you very much for the helpful and meaningful suggestions and comments from the Scientific Editor and the Reviewer.

We answer your questions and comments below.

All the changes in the article are highlighted in yellow.

Due to his help in the additional experiments, we listed Dr. Gábor Petővári as a co-author.

We hope that, based on the answers and the results of the additional studies we have conducted, our manuscript is now acceptable for publication in the Plos One journal.

Yours Sincerely,

Ferenc Sipos MD, PhD

**Answers to Scientific Editor**

Thank you for your precious advices and meaningful suggestions.

Here are our answers to your comments:

***1) The main drawback of the study is the lack of autophagic flux assessment. While the Methods section contains the "Assessing autophagic flux" part, it describes how the cells were treated with chloroquine, but not how the flux was actually assessed. Since the autophagic flux was also not determined in their previous publication (Pathol Oncol Res 2019;25:1505), the authors are advised to perform the flux measurement experiments (e.g. immunoblot assessment of LC3 conversion in the presence of lysosomal inhibitors and/or levels of autophagy-selective targets such as SQSTM1/p62) in accordance with the current guidelines (Autophagy 2021;17:1-382. doi: 10.1080/15548627.2020.1797280).***

**Our answer:** The term „assessing autophagic flux” was incorrectly used. However, references in the literature also refer to chloroquine (CQ) as an inhibitor for autophagic flux determination. CQ as an anti-inflammatory substance, is the most commonly used autophagy completion inhibitory drug to ascertain autophagic flux because of its suitability in vivo [Moulis M, Vindis C. Methods for Measuring Autophagy in Mice. Cells. 2017;6(2):14. doi:10.3390/cells6020014].

CQ has long been a known and used functional inhibitor in the study of the autophagy process [Klionsky DJ, Abdel-Aziz AK, Abdelfatah S, et al. Guidelines for the use and interpretation of assays for monitoring autophagy (4th edition)1. Autophagy. 2021;17(1):1-382. doi:10.1080/15548627.2020.1797280]. CQ initially initiates the process of autophagy, but eventually inhibits its final step. Because the short-, medium-, and long-term effects [Klaschik S et al. Journal of Leukocyte Biology, 2009; 85(5): 788–795.; Ito SI et al. Infection and Immunity, 2004; 72(6): 3543–3548.; Verthelyi D et al. The Journal of Immunology 2003; 170(9): 4717–4723.; Klaschik S et al. Molecular Immunology 2010, 47(6): 1317–1324.] of DNA treatment-TLR9 interaction were studied together, cell experiments were performed with a 72h long incubation. During this time, CQ also has an inhibitory effect on autophagy. As it is an inexpensive drug used in human therapy, it also advocated its use to inhibit autophagy in this study.

We performed SQSTM1/p62 and LC3B WES Simple Western blot experiments for the combined treatment settings. The results have been inserted to the manuscript.

***2) The doubling time of HT29 cells is approx. 24 h. Therefore, it is unclear how the proliferation could be suppressed by almost 90% (Table 2, gDC treatment) after only 4 h of incubation? Since the authors observed that some treatments caused cells to "disintegrate", the observed suppression is more likely due to cell death. This issue should be clarified.***

**Our answer:** The 4-hour AlamarBlue assay was performed in the treated (e.g., gDC) cell groups after a treatment period of 72 hours. And compared to control untreated cells, the cell proliferation was reduced by 90% (tested within 76 hours /72h + 4h/). The disintegration of the cell structure can also indicate apoptosis, that is why we investigated the gene expression alterations related to different modes of apoptosis as well.

***3) Either Table 2 or Fig. 1 should be omitted, as they present the same data.***

**Our answer:** The table and the figure both display part of the same information. The table is more comprehensive. However, because the figure makes it easier to depict differences in the treatment groups, we've decided to keep it as a supplementary figure.

***4) Table 2/Fig. 1: ANOVA should be used to compare different treatments where appropriate, and the significant differences could be indicated by compact letter display. Also, the authors should state which post-hoc test (if any) was used for multiple comparisons.***

**Our answer:** In the case of cell proliferative capacity and survival experiments, statistical analysis with one-way ANOVA and Tukey HSD test was performed as well. In addition to comparison within control sample groups (K, O, D, C, Kg, Kf, Km), the modified DNA + inhibitor combination groups (i.e., gO, gD, gC, gOD, gDC; fO, fD, fC, fOD, fDC; and mO, mD, mC, mOD, mDC) were compared to absolute control cells (i.e., K).The results of the ANOVA and Tukey HSD analyses now are available as a supplementary file.

***5) Fig. 2 and Fig. 3: Gene expression data should be presented as mean ± SD and the observed differences statistically analyzed.***

**Our answer:** Comparison of the NanoString nCounter gene expression system with microarrays and TaqMan PCR demonstrated that the nCounter system is more sensitive than microarrays and similar in sensitivity to real-time PCR [Geiss et al., 2008, DOI: 10.1038/nbt1385.; Goytain et al., 2020, DOI: 10.1007/978-1-4939-9904-0_10]. NanoString nCounter system is a preferred method when input the total RNA is partially degraded or not in an ideal quality [Tachibana, 2015, DOI: 10.1126/science.349.6247.544]. The advantages of nCounter system over the RT-qPCR are that the nCounter system is directly count or quantitate the gene expression levels without involving PCR steps, and only 100 ng of total RNA is needed to accurately measure the expression of a few hundred targeted genes per sample [Geiss et al., 2008, DOI: 10.1038/nbt1385.; Kulkarni, 2011, DOI: 10.1002/0471142727.mb25b10s94]. Moreover, nCounter analysis system is a reliable method to validate expression data that were generated from not-an-ideal-quality samples [Othman et al. 2018, DOI: 10.21894/jopr.2018.0009]. Taking into account the above long-known facts, we used NanoString technology.

Nevertheless, taking into account the MIQE guidelines [Bustin et al., 2009, DOI: 10.1373/clinchem.2008.112797] and dMIQE guidelines [dMIQE group, 2020, doi: 10.1093/clinchem/hvaa125.; doi: 10.1093/clinchem/hvaa219.], we have performed a Taqman RT-qPCR validation of the used NanoString Custom platform. The Taqman results validated the NanoString results, as it can be seen as a supplementary file.

***6) The concentration of chloroquine (1 μM) seems unusually low for autophagy inhibition. The authors' own data show 3 ± 1 vs. 4 ± 1.5 autophagic vesicles in control vs. chloroquine-treated cells, indicating no significant inhibition of basal autophagy, and the similar results (no additional accumulation of autophagic vesicles and thus no autophagy inhibition) were observed when choloroquine was combined with other treatments. This makes difficult any interpretation of the role autophagy could possibly have in the observed effects. While I am aware of the extent of required additional experimentation, the experiments should be repeated with the chloroquine concentration that inhibits autophagy. An alternative possibility would be to describe chloroquine effects as autophagy-independent, in which case the authors must provide a rationale for using the drug at the concentration unable to block autophagy.***

**Our answer:** The concentration of 1 μM was incorrectly indicated. CQ was used at a concentration of 10 μM. We apologize for the error.

This concentration is also lower than that used in short-term (4h to 24h long) treatments to inhibit autophagy [BMC Cancer. 2010 Jul 15;10:370. doi: 10.1186/1471-2407-10-370. PMID: 20630104; APMIS. 2012 Jul;120(7):597-604. doi: 10.1111/j.1600-0463.2012.02876.x. PMID: 22716215.]. The core of our experiment, however, was designed to account for the acute, subacute, and late activation durations of TLR9 DNA binding, which translates to a 72-hour treatment time [Klaschik S et al. Journal of Leukocyte Biology, 2009; 85(5): 788–795.; Ito SI et al. Infection and Immunity, 2004; 72(6): 3543–3548.; Verthelyi D et al. The Journal of Immunology 2003; 170(9): 4717–4723.; Klaschik S et al. Molecular Immunology 2010, 47(6): 1317–1324.]. Based on our preliminary experiments, CQ treatment at a concentration of 10 μM effectively inhibited autophagy without significantly affecting the proliferation of HT29 cells after 72h of incubation. Here the proliferation inhibitory effect of different concentrations of CQ can be seen in HT29 and DLD1 colon adenocarcinoma cells.

***7) Chloroquine has been reported to kill cancer cells independently of autophagy inhibition (Autophagy 2017;13:955, Graefes Arch Clin Exp Ophthalmol 2015;253:2309, Eur J Pharmacol 2019;859:172540, Pharm Res 2012;29:2249), which should be acknowledged and discussed.***

**Our answer:** we have been acknowledged and discussed this aspect of CQ treatment. Thank you for this important comment.

***8) The effects of multiple treatments on cell proliferation should be explained more clearly. Namely, the authors seem to suggest that the anti-proliferative effect of self-DNA was partly mediated by autophagy induction, and that cell proliferation in various treatment is inversely correlated with autophagy. Therefore, the increase in autophagy gene expression and further block in cell proliferation by chloroquine is somewhat counterintuitive and requires a thorough explanation.***

**Our answer:** Although all types of modified self-DNA treatment increased the expression of autophagy-related genes, cell proliferation was only enhanced after fDNA administration. The combination of DISU with DNA resulted in the accumulation of p62 and LC3B proteins, which indicate autophagy inhibition. With respect to cell proliferation, the combined effect of DISU and DNA was the opposite to that of DNA alone. This may be due to the complex two-way relationship between recently discovered autophagy master regulatory kinases (incl. HGFR) and autophagy-related proteins [DOI: 10.1080/15384101.2017.1372548; DOI: 10.1038/ncomms11942; DOI: 10.1038/cdd.2017.41].

Essentially, inhibiting autophagy and intracellular accumulation of p62 protein stimulates cell proliferation in tumor cells. Nevertheless, accumulation of p62 (and LC3B) in HT29 cells and CRC tissue (suggesting impaired autophagy alone) resulted in decreased tumor cell survival and a favorable prognosis in patients. All of this suggests that there may be a link between autophagy inhibition and decreased cell proliferation. In any case, we combined autophagy inhibition with gDNA and DISU in our case, and the combined effect was an inhibition of cell proliferation. Autophagy mediated by the HGFR-mTOR-ULK1 cascade may also contribute to this [DOI: 10.1038/s41419-019-1314-x, DOI: 10.1016/j.bbrc.2012.12.120, DOI: 10.3389/fonc.2021.638701, DOI: 10.18632/oncotarget.17554].

The highest extent of cell proliferation was found when mDNA, ODN2088 and DISU were co-administered. In this case, decreased expression of both canonical and non-canonical HGFR signaling pathways was observed. The down-regulation of autophagy related genes, however, was also present. Though autophagy induction is known to promote cell proliferation, it was demonstrated that genetic silencing of key autophagy proteins (e.g., Beclin 1, Ambra 1) in mice can lead to increased cell proliferation [DOI: 10.1016/j.febslet.2010.01.009]. The possibility that the combination of treatments used had an epigenetic effect cannot be ruled out.

***9) Lines 308-310: What is the difference between "moderate upregulation" and "mild overexpression"? If there is none, please rewrite the sentence.***

**Our answer:** we have rephrased these sentences, according to your suggestions.

***10) The introductory sentence of the Discussion section should be revised to increase clarity. "In this study, we attempted to answer how HGFR inhibition modulates the effect of tumor-derived self-DNA on TLR9 signaling and autophagy response by examining the metabolic activity and proliferation of HT29 colon cancer cells." One cannot examine the effects on autophagy by measuring metabolic activity and proliferation.***

**Our answer:** we have rephrased the first paragraph according to your suggestions.

We trust that, based on our responses and the results of additional studies conducted, you will consider the manuscript suitable for publication.

**Answers to Reviewer 1**

Thank you for your valuable review, helpful advice, and meaningful suggestions.

Here are our answers to your comments:

***1. „in my opinion author can prove the mechanism in another colon cancer cell line with an inducible or overexpressed system beside HT29 cells with constitutive expression of TLR9.”***

**Our answer:** For the combined study of self-DNA-induced TLR9 activation, autophagy response, and HGFR signaling, the cell lines tested must meet specific conditions. We decided to use HT29 colon adenocarcinoma cells, because of several aspects. There is a basal TLR9 expression in HT29 cells, which is essential for induction with self-DNA. Since DNA treatment via TLR9 can exert its effects in MyD88-dependent way, it is also important that the MyD88-dependent TLR signaling pathway is intact in these cells. HGFR expression in HT29 cells is present. Also, in HT29 cells, HGF expression can be detected, which is essential for both autocrine activation of HGFR signaling and studying the effect of HGFR inhibition. In HT29 cells TLR and autophagy-mediated HGFR cross-activation is also present. Nevertheless, the HT29 cells adequately represent sporadic colon cancers [Wang C, Jet al. PLoS One. 2016; 11: e0152177; Takeuchi H, et al. Clin Cancer Res 2003; 9: 1480-1488; Ling C, et al. Hum Gene Ther. 2010; 21: 1741-7; Bell ES, et al. Cell Rep 2019; 29: 4053-4068.e6; Nowakowska M, et al. Tumour Biol. 2014; 35: 9291-301].

We are examining other cell lines, especially for modeling CRC metastasis formation.

***2. The authors should execute more molecular biology based experiments such as rtPCR or western blot of most important genes or proteins respectively to show the signaling pathway and metabolic activity based mechanism.***

**Our answer:** As recommended, additional WES Simple Western blot and Taqman RT-PCR studies were performed, which consistently confirmed the results of the other methods. The results of the new methods were also included in the discussion.

***3. In the methodology part, some lines are exactly imitated from Ref.21. The authors should take care to avoid plagiarism, specially “HT-29 cell treatment”, “C-MET, TLR9 and autophagy immunocytochemistry”, and “Transmission electron microscopy for evaluation of autophagy” portion.***

**Our answers:** The indicated description of methodologies has been rephrased.

***4. The authors may also include the following reference on Autophagy in the introduction section along with 13, i) A novel betulinic acid analogue ascertains an antagonistic mechanism between autophagy and proteosomal degradation pathway in human colon carcinoma cells (HT29). BMC Cancer, 2016, 16:23, 1-19.(DOI:*** [***https://doi.org/10.1186/s12885-016-2055-1***](https://doi.org/10.1186/s12885-016-2055-1)***)***

**Our answer:** The suggested reference has been inserted.

***5. The authors may also include the following reference on Aptamer targeted therapy for colon cancer, in the introduction section along with 19, ii) Aptamer-Conjugated Apigenin Nanoparticles To Target Colorectal Carcinoma: A Promising Safe Alternative of Colorectal Cancer Chemotherapy, ACS Appl. Bio Mater, 2018. (DOI: 10.1021/acsabm.8b00441)***

**Our answer:** The suggested reference has been inserted.

We are confident that you will find the manuscript suitable for publication based on our responses and the results of additional studies.
